# Supplementary material for: Morningness–eveningness assessment from mobile phone communication analysis
Source: Sci Rep. 2021 Jul 16;11:14606. doi: 10.1038/s41598-021-93799-0 (PMC8285513; doi:10.1038/s41598-021-93799-0)
Supplement: Supplementary file 1 — Supplementary Information. [file 41598_2021_93799_MOESM1_ESM.pdf]

Morningness-eveningness assessment from mobile phone communication analysis

Chandreyee Roy,[1,*] Kunal Bhattacharya,[1,2] Daniel Monsivais,[1] Robin I.M. Dunbar,[3] and Kimmo Kaski[1,4]

[1] *Department of Computer Science, Aalto University School of Science, Espoo, Finland*

[2] *Department of Industrial Engineering and Management, Aalto University School of Science, Espoo, Finland*

[3] *Department of Experimental Psychology, University of Oxford, Oxford, UK*

[4] *The Alan Turing Institute, London, UK*

S1. PRINCIPAL COMPONENT ANALYSIS OF THE MID-SLEEP TIMES

We have performed a principal component analysis on the mid-sleep times of the vector $\vec{T}_{mid} = \{T_{mid}^{Weekday}, T_{mid}^{Friday}, T_{mid}^{Saturday}, T_{mid}^{Sunday}\}$ as discussed in the main text. The loadings of the T_{mid}^{d} has been summarized in Table S1. Since all the loadings have negative values we use **-PC1** as a convention to study the chronotypes from $\vec{T}_{mid}$. The reversal of sign does not affect the results in the case of PCA because the principal axis is rotated arbitrarily to get the best fit of the data. Moreover, we have found that the correlation between the PC1 and g chronotype (discussed in details in later sections) have a negative value of -0.73. Thus we have reversed the sign for non regressed values of T_{mid}^{d} to maintain the same conventions for all chronotypes.

	$T_{mid}^{Weekday}$	T_{mid}^{Friday}	$T_{mid}^{Saturday}$	T_{mid}^{Sunday}
PC1	-0.47	-0.50	-0.54	-0.50

TABLE S1.

S2. REGRESSION OF THE MID-SLEEP TIMES

To remove the effect of the East-West sun progression, we performed a regression on the T_{mid}^{d} values for all d days with latitude and longitude as independent variables. The values of the intercept, coefficients of latitude and longitude have been enumerated in Table S2 along with their p values.

* chandreyee.roy@aalto.fi

	Intercept	p value	Longitude	p value	Latitude	p value
$T_{mid}^{Weekday}$	35.45	< 0.0001	-0.12	< 0.0001	0.02	0.005
T_{mid}^{Friday}	38.14	< 0.0001	-0.14	< 0.0001	-0.01	0.114
$T_{mid}^{Saturday}$	38.43	< 0.0001	-0.16	< 0.0001	0.0	0.786
T_{mid}^{Sunday}	35.64	< 0.0001	-0.11	< 0.0001	0.04	0.0004

TABLE S2.

S3. EXPLORATORY FACTOR ANALYSIS

An exploratory factor analysis performed on the morning and evening activities of the dataset reveals that there are two underlying constructs or latent factors in the data. They are characterized by morning behaviour and evening behaviour of the individuals and the loadings of these factors on the observables i.e. the calling activities of the individuals have been summarised in Table S3. The boxes coloured in blue shows the high values of the MB on morning activities only and EB on evening activities clearly illustrating the two distinct factors arising from factor analysis.

	Morning Behaviour (MB)	Evening Behaviour (EB)	communality
MA_{Work}	0.90	-0.02	0.79
MA_{Fri}	0.51	0.17	0.34
MA_{Sat}	0.45	0.16	0.27
MA_{Sun}	0.81	-0.03	0.65
EA_{Week}	0.03	0.84	0.72
EA_{Fri}	-0.03	0.84	0.69
EA_{Sat}	0.01	0.57	0.33
EA_{Sun}	-0.04	0.49	0.23

TABLE S3.

S4. EXPLORATORY BIFACTOR ANALYSIS

We perform a bifactor analysis to compute g chronotype described in the main text. The loadings of all the factors, g chronotype, F1* and F2*, factors have been summarised in Table S4. The general factor g loads directly onto all the calling activities of the individuals, thus computing a score that

	g chronotype	F1*	F2*
MA_{Week}	0.49	0.74	
MA_{Fri}	0.37	0.42	
MA_{Sat}	0.34	0.38	
MA_{Sun}	0.43	0.68	
EA_{Week}	0.49		0.70
EA_{Fri}	0.45		0.70
EA_{Sat}	0.33		0.48
EA_{Sun}	0.25		0.40

TABLE S4.

is used to identify their chronotypes. F1* and F2* are group factors that load separately onto the morning and the evening activities.

S5. REGRESSION OF THE CALLING ACTIVITIES

We again carry out a regression on the morning ($\{MA_d\}$) and evening activities ($\{EA_d\}$) of the individuals for all d days of the week to remove the geographical effect using longitude and latitude as independent variables. In Table S5 we have summarised the results obtained from computing the regression along with the p values.

	Intercept	p value	Longitude	p value	Latitude	p value
MA_{Week}	59.53	< 0.0001	-0.06	< 0.0001	0.08	< 0.0001
MA_{Fri}	61.00	< 0.0001	-0.10	< 0.0001	0.05	< 0.0001
MA_{Sat}	60.37	< 0.0001	-0.13	< 0.0001	0.07	< 0.0001
MA_{Sun}	59.22	< 0.0001	-0.05	< 0.0001	0.08	< 0.0001
EA_{Week}	17.36	< 0.0001	-0.10	< 0.0001	-0.15	< 0.0001
EA_{Fri}	18.02	< 0.0001	-0.11	< 0.0001	-0.16	< 0.0001
EA_{Sat}	17.87	< 0.0001	-0.08	< 0.0001	-0.14	< 0.0001
EA_{Sun}	17.08	< 0.0001	-0.05	< 0.0001	-0.13	< 0.0001

TABLE S5.

S6. FACTOR SCORES OF THE RESIDUALS COMPUTED AFTER REGRESSION

After carrying out the regression on MAs and EAs we perform an exploratory analysis and bifactor analysis on the residuals obtained. We show the variation of the factor scores of the morning and evening behaviour, the general g chronotype and m chronotype (see section S7) for the 5 regions (longitudinal bands). This plot is computed after an EFA and EBA was done on the residuals to show that we have removed the effect of the East-West progression in the data. Figure S1 shows a summary of the factor scores in the form of a box-plot that includes all the values within the range of the 25th and 75th percentile and the end of the whiskers represent the maximum and the minimum scores excluding outliers. The horizontal lines inside the boxes represent the median of the scores in each region.

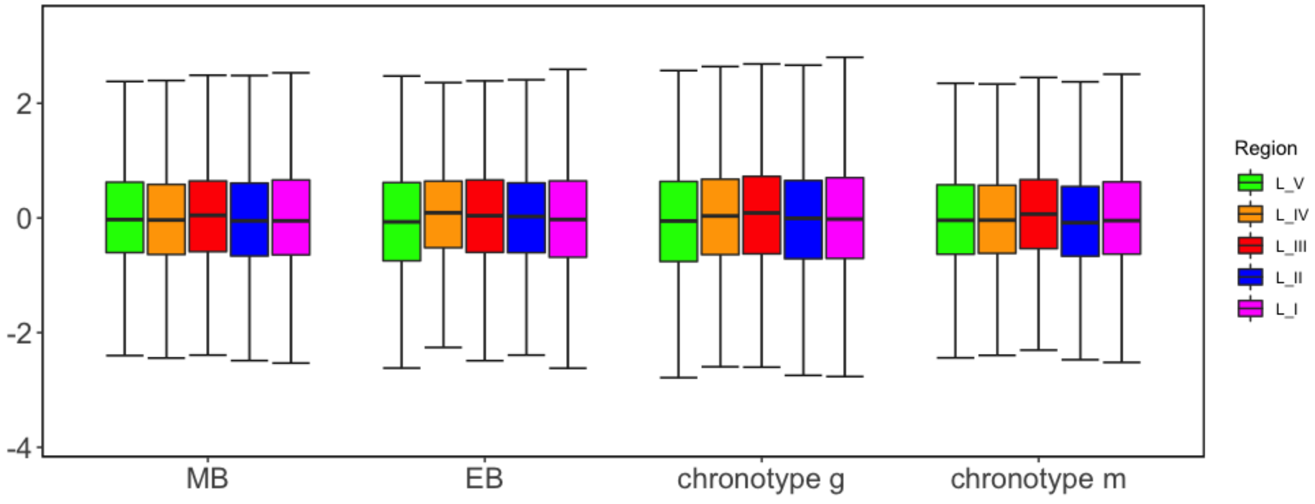

FIG. S1.

S7. THE M CHRONOTYPE AND ITS VARIATION WITH AGE AND GENDER

Finally, we have considered all the T^d_{mid} values for all d days of the week. This model has a unidimensionality score is 0.97 which implies that there is only one underlying factor. An EFA carried out on this model also shows that there is one latent factor that can be used as indicator of the chronotype (m chronotype) of an individual as shown in Figure S2. The two chronotypes: g and m have a strong correlation (0.78) and both can used to determine the morningness or eveningness of a user. The colour blue has been used males and red for females.

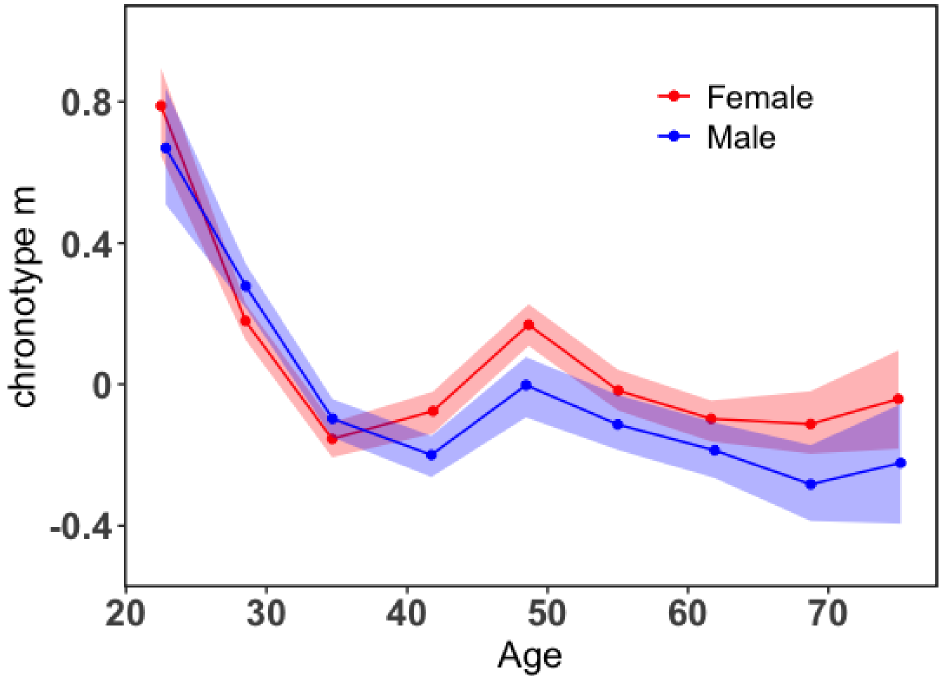

FIG. S2.
